# Healthier Smile: The Role of Diet and Nutrition in the Prevention and Therapy of Caries, Gingivitis, and Periodontitis

**DOI:** 10.3390/nu15204319

**Published:** 2023-10-10

**Authors:** Johan Peter Woelber, Kirstin Vach

**Affiliations:** 1 Policlinic of Operative Dentistry, Periodontology, and Pediatric Dentistry, Medical Faculty Carl Gustav Carus, Technische Universität Dresden, Fetscherstraße 74, 01307 Dresden, Germany; 2 Department of Conservative Dentistry, Periodontology and Preventive Dentistry, Hannover Medical School, Carl-Neuberg-Str. 1, 30625 Hannover, Germany; 3 Institute of Medical Biometry and Statistics, Faculty of Medicine, University of Freiburg, Stefan-Meier-Str. 26, 79104 Freiburg, Germany

Although oral hygiene and fluorides have a significant impact on people’s oral health, we must not forget that the causes of oral diseases are often related to malnutrition and other unhealthy behavioral factors, such as smoking, being sedentary, and chronic stress. While dental caries is clearly a diet-related disease [1,2], there is also growing evidence that gingivitis and periodontitis are greatly influenced by diet [3,4,5,6,7,8,9]. A peculiarity in this context is that malnutrition with far too much sugar and pro-inflammatory fats and a lack of fiber and micronutrients (which is also what the average Western diet represents) shows up extremely quickly in oral diseases [5,10,11,12]—much quicker than other diseases also caused by Western diets, such as obesity, diabetes, or atherosclerosis. This, in turn, presents a great opportunity for dentists and physicians to use nutritional dentistry to initiate healthier diets early on, before other secondary diseases manifest themselves—an opportunity for which solely plaque control and fluorides are inadequate [4,13]. However, while broad evidence already exists for plaque control and fluorides, more evidence is needed for the efficacy of nutritional dentistry.

With this background, we are honored to support this process with this Special Issue “Healthier smile: The role of diet and nutrition in the prevention and therapy of oral diseases”. It includes five studies and a systematic review:

Feldens et al. [14] investigated the long-term impact of breastfeeding and pacifier use on increased overjet in permanent dentition in a prospective cohort (n = 214), from birth to 12 years of age. The authors concluded that breastfeeding protected half of the cohort from increased overjet in adolescence through reducing pacifier use. This study highlights the importance of a natural diet from the beginning of life.

Staufenbiel et al. [15] investigated the influence of nutrition and physical activity on the course of experimental gingivitis. They analyzed the data of thirty-nine non-smoking periodontally healthy participants and concluded that both an anti-inflammatory diet (measured with the dietary inflammatory index) and physical activity, or, even better, a combination of both, were able to reduce the amount of bleeding on probing (BOP).

In a cross-sectional study of n = 5642 participants of the Hamburg City Health Study (HCHS), Lieske et al. [16] analyzed the association of different inflammatory properties of diet in relation to periodontitis. They found that a higher anti-inflammatory diet score was significantly associated with lower odds of periodontal disease.

In another cross-sectional study, n = 537 participants from southern Brazil (1982 Pelotas Birth Cohort) were analyzed by Cassiano et al. [17] with regard to their food consumption and the occurrence of periodontitis. The authors found a significant association between moderate and severe periodontitis and processed/ultra-processed food consumption. 

To date, it is largely unknown how to predict which participants and their corresponding microbiomes will respond to dietary changes. To provide further evidence on this issue, Vach et al. [18] analyzed the changes in the microbiome of 11 different participants in different dietary phases. The developed method was suitable for identifying typical and atypical responders, even in small datasets, and could be used in the future for targeted individualized dietary advice.

In a systematic review, Woelber et al. [19] investigated the usefulness of dietary and nutraceutical adjuncts in non-surgical periodontal therapy. A significant positive effect on periodontal outcome parameters (PPD, bleeding on probing) was found for vitamin E, chicory extract, juice powder, green tea, and oolong tea. 

Summarizing the evidence presented, it seems increasingly clear that periodontal inflammation and the extent of periodontitis can be strongly influenced by diet. This knowledge can be used in both prevention and adjunctive therapy. Since the occurrence of a modern Western diet was not foreseen in human evolution, nutrition must be addressed accordingly for the causal prevention and therapy of oral diseases and, if necessary, therapeutically influenced.
